# Genotype variation of *ACE* and *ACE2* genes affects the severity of COVID-19 patients

**DOI:** 10.1186/s13104-023-06483-z

**Published:** 2023-09-04

**Authors:** Ingrid Faustine, Deli Marteka, Amarila Malik, Eko Supriyanto, Nadia F. Syafhan

**Affiliations:** 1 Faculty of Pharmacy, Universitas Indonesia, Depok, 16424 West Java Indonesia; 2 Division of Pharmaceutical Microbiology and Biotechnology, Faculty of Pharmacy, Universitas Indonesia, Depok, 16424 West Java Indonesia; 3 Department of Biomedical Engineering & Health Science, Universiti Teknologi Malaysia, Johor Bahru, Johor, 81310 Malaysia; 4 Division of Clinical Pharmacy, Faculty of Pharmacy, Universitas Indonesia, Depok, 16424 West Java Indonesia; 5 Universitas Indonesia Hospital, Jl. Prof. Dr. Bahder Djohan, Pondok Cina, Depok, 16424 West Java Indonesia; 6 Department of Pharmacy, Faculty of Mathematics and Natural Sciences, Tadulako University, Palu, 94148 Central Sulawesi Indonesia

**Keywords:** *ACE* gene, *ACE2* gene, rs4331, rs2074192, COVID-19, Indonesian population, Hypertension comorbide, rhAmp SNP genotyping

## Abstract

**Objective:**

Genetic polymorphisms in *ACE* and *ACE2* genes are involved in the RAS regulation of blood pressure and their activity may confer susceptibility to hypertension. In addition, they may play a role in SARS-CoV-2 pathogenesis and the severity of COVID-19. This study aims to determine the effect of genetic variations in the *ACE* (rs4331) and *ACE2* (rs2074192) genes with hypertension comorbidity on the severity of COVID-19 in the Indonesian population.

**Result:**

186 patients were enrolled and assigned into the COVID-19 group (n = 95) and non-COVID-19 group (n = 91) in this cross-sectional study. GG genotype frequency was dominant in *ACE* gene, but there were no significant differences between the groups (p = 0.163). The two groups had a significant difference (p = 0.000) for the CC genotype frequency (0,37 vs. 0.01) in the *ACE2* gene. The proportion of women with COVID-19 is higher (51%), but men with hypertension had more severe symptoms (44%). Men with hypertension comorbidity, GG (*ACE*), and TT (*ACE2*) genotypes tended to have moderate-to-severe symptoms (25%). Similarly, women with hypertension as well as GG and CT genotypes tended to have moderate-to-severe symptoms (21%). We conclude that hypertension and mutations in the *ACE* (rs4331) and *ACE2* (rs2074192) genes affect the severity of COVID-19.

**Supplementary Information:**

The online version contains supplementary material available at 10.1186/s13104-023-06483-z.

## Introduction

To date, there have been 6.8 million cases of COVID-19 in Indonesia; 50.4% of them are women, and 48% have hypertension comorbidity, with a mortality rate of 2.4% [1], which is greater than the global mortality rate of 0.9% (May 2023) [2]. Most patients infected with SARS-CoV-2 were asymptomatic, but of the patients requiring hospitalization, 86% were classified as moderate and severe, with 17% of patients died [3].

The Renin-Angiotensin System (RAS) is a blood pressure regulatory pathways. It is known to be involved in the pathogenesis of COVID-19, in which SARS-CoV-2 uses Angiotensin-Converting Enzyme-2 (ACE2) as a receptor-binding agent to enter the cell [4]. Decreased ACE2 bioavailability and increased ACE activity level can increase the activity of Angiotensin-II (AngII) in RAS and may lead to COVID-19-induced inflammation and lung injury [5].

Genetic polymorphisms in *ACE* and *ACE2* genes have been shown to confer susceptibility to hypertension [6, 7]. In the pathogenesis of COVID-19, *ACE2* gene polymorphism located in X chromosome was postulated to cause a higher prevalence of COVID-19 in men [8]. Two rs numbers that were included in SNPs with a MAF greater than 10%, i.e., rs4331 and rs1799752 (I/D), as reported by Chung (2013) [9], were chosen to be analyzed in this study. However, our previous study in Indonesian population with COVID-19, which were type II dominant, showed inconsistent and inconclusive results (unpublished observations). Another study showed that the rs4331 polymorphisms were in linkage disequilibrium with the *ACE* I/D polymorphisms and were readily accessible by the rhAmp assay [10]. Therefore, we attempted to provide evidence of the effect of genetic variation of the rs4331 *ACE* gene and rs2074192 *ACE2* gene on the severity of COVID-19 in COVID-19 patients with hypertension comorbidity.

## Materials and methods

### Design and study participant

In this cross-sectional study, we collected participants from 18-year-old patients from health facilities located in Lahat District in South Sumatra Province, located in the Western part of Indonesia, and the city of Palu in Central Sulawesi Province, located in the Eastern part Indonesia, with a minimum sample size calculated using a predetermined formula. We collected anthropometric and clinical data from 95 COVID-19 participants who were positive for SARS-CoV-2 (nose or throat swab real-time-polymerase chain reaction (rt-PCR) test) who received treatment in a hospital or underwent self-isolation under the monitoring of a health facility. The COVID-19 severity category is based on our preliminary research, which refers to the NIH guidelines [3, 11]. We also selected 91 non-COVID-19 populations from outpatient clinics without matching age or sex for the baseline group. Another inclusion criterion is that the participants should have no previous history of COVID-19 diagnosis based on a positive rtPCR test for the SARS-CoV-2 virus. History of hypertension comorbidity was obtained from the participant’s medical records.

### Genotyping of SNP by rhAmp SNP Genotyping

Genomic DNA was extracted from whole blood using the QIAamp DNA Blood Mini Kit (QIAGEN, Hilden, Germany) and measured using a NanoDrop™ One Microvolume UV–Vis Spectrophotometer (Thermo Fisher Scientific, Waltham, MA). DNA was diluted to a concentration of 2 ng/µL. One *ACE* SNP (rs4331; A/G) Exon 15 and one *ACE2* SNP (rs2074192; C/T) intron 16 were identified by the rhAmp SNP genotyping method according to the rhAmp SNP protocol [12]. The PCR conditions were as follows: enzyme activation at 95 °C for 10 min, followed by 40 cycles of denaturation at 95 °C for 10 s, annealing at 60 °C for 30 s, extension at 68 °C for 20 s, ending with heat inactivation at 99.9 °C for 15 min. Quality control is always performed during laboratory work, where gBlock and NTC are used as positive and negative controls, respectively. Amplification was performed using AriaMx RT-PCR (Agilent, Santa Clara, US). AriaMx software was used to generate allele calls and allele discrimination plots automatically.

### Statistical analysis

Population genetic data was analyzed using the Hardy-Weinberg equilibrium principle with the online software SNPStats (https://www.snpstats.net/). Categorical variables were expressed as amounts and percentages. Differences between groups were evaluated using Fisher’s exact or Chi-square test, depending on sample size and multiple comparison corrections with Bonferroni correction. We used SPSS version 21.0 (IBM Corp., Armonk, NY, USA) for all statistical analysis. We used two-tailed *p*-values in our analysis with a *p* < 0.05 level of significance.

## Result

### Distribution of genetic variation of *ACE* and *ACE2* genes

**Fig. 1:**
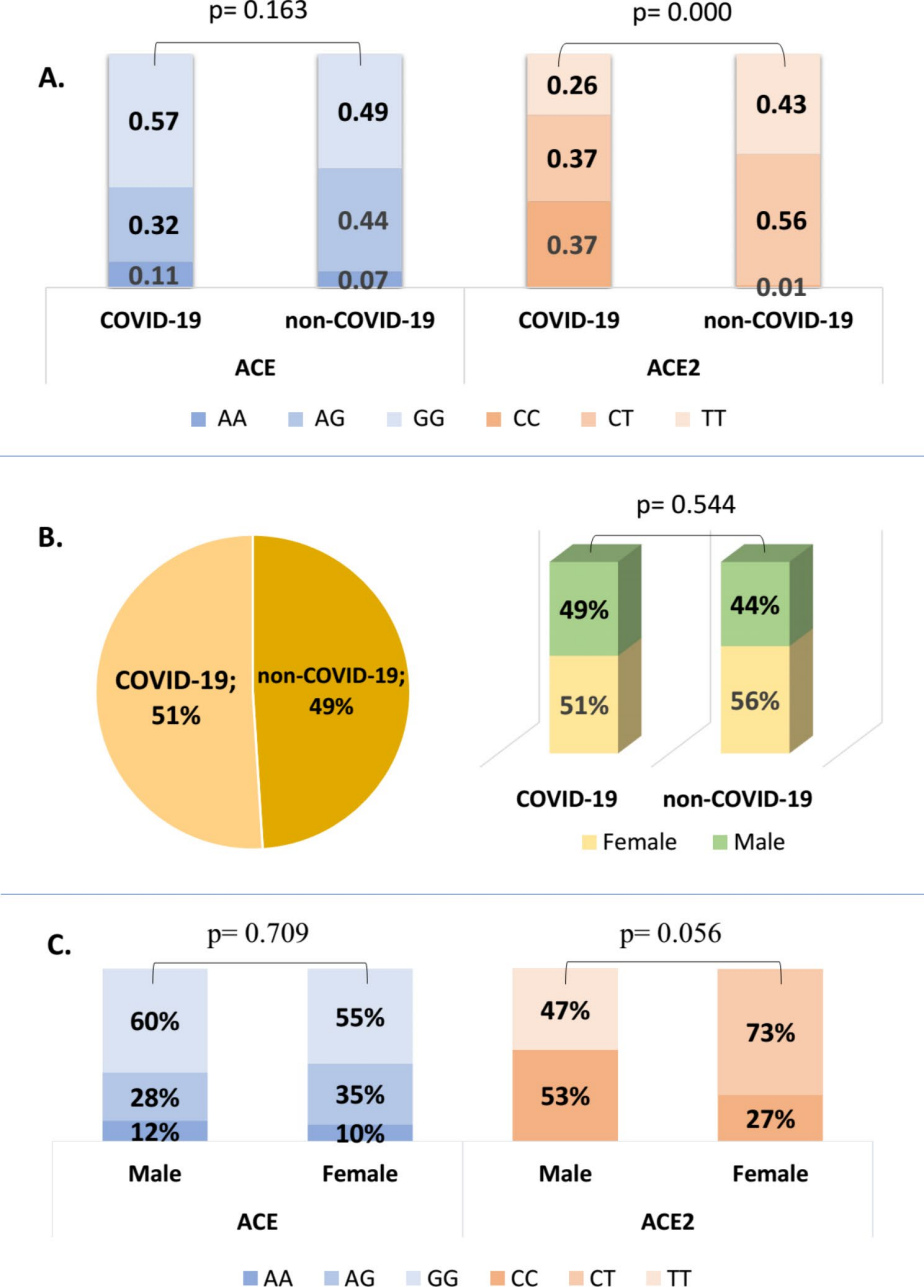
Genotype distribution of *ACE* and *ACE2* genes in COVID-19. Figure 1 A Genotype frequency of *ACE* and *ACE2* in the non-COVID-19 and COVID-19 groups. Figure 1B shows the percentages of COVID-19 and non-COVID-19 groups, and percentages of sex of each group. Figure 1 C shows the percentage of each genotype by sex in the COVID-19 group

One-hundred eighty-six participants were recruited and divided into COVID-19 groups and non-COVID-19 groups. The frequency of the GG genotype *ACE* gene rs4331 and CT genotype *ACE2* gene rs2074192 were dominant in both the COVID-19 and non-COVID-19 groups (Fig. 1A). No significant difference was observed in the proportion of *ACE* gene genotypes (p = 0.163), whereas in the *ACE2* gene, a significant difference was observed in the proportion of genotypes (p = 0.000), in which CC genotype poses more risk for COVID-19 compared to the non-COVID-19. Of the total sample, there were 49% of non-COVID-19 patients and 51% of COVID-19 patients, but based on sex, there was no significant difference between the two groups (p = 0.544) (Fig. 1B). When comparing sexes, the percentage of GG genotypes *ACE* gene in men was found to higher than in women. In contrast, in the *ACE2* gene, the TT genotype was found more in men (2%) and absent in women, and women in the COVID-19 group were more likely to have the CT genotype (Fig. 1C).

### Association of clinical characteristics with the severity of COVID-19

We then analyzed the data to determine the associations between clinical characteristics and the severity by sex in the COVID-19 group. As shown in Table 1, there were no differences in terms of age, hypertension comorbid, BMI, type of care, and length of stay for both male and female with mild and moderate-to-severe COVID-19 (p > 0.05). However, of the eleven symptoms recorded (Suppl. Table 1), we found that anosmia and vomiting were dominant in women with moderate-to-severe COVID-19 (p < 0.05).

**Table 1:** Clinical characteristics of severity COVID-19 by sex

Variables	Mild		p-value	Moderate-Severe		p-value
	Male (n = 20)	Female (n = 20)		Male (n = 27)	Female (n = 28)	
Age (y)			0.403			0.435
18–39	6 (30%)	10 (50%)		8 (30%)	10 (36%)	
40–59	12 (60%)	8 (40%)		14 (52%)	16 (57%)	
≥ 60	2 (10%)	2 (10%)		5 (18%)	2 (7%)	
Hypertension Comorbidity	3 (15%)	4 (20%)	0.500	12 (44%)	9 (32%)	0.509
BMI (Overweight-to-Obese)	9 (45%)	11 (55%)	0.752	16 (36%)	16 (57%)	1.000
Symptoms						
Anosmia	12 (60%)	10 (50%)	0.751	1 (4%)	14 (50%)	0.000
Vomiting	4 (20%)	4 (20%)	0.653	1 (4%)	8 (29%)	0.014
Treatment						
Hospital Admission	9 (45%)	4 (20%)	0.088	27 (100%)	27 (96%)	0.509
Self-isolation	11 (55%)	16 (80%)		0 (0%)	1 (4%)	
LoS (> 13 days)	10 (50%)	15 (75%)	0.191	4 (15%)	10 (36%)	0.142

BMI (Body Mass Index), LoS (Length of Stay)

### Genetic variation of *ACE* and *ACE2* on comorbidities, sex, and severity of COVID-19

**Fig. 2:**
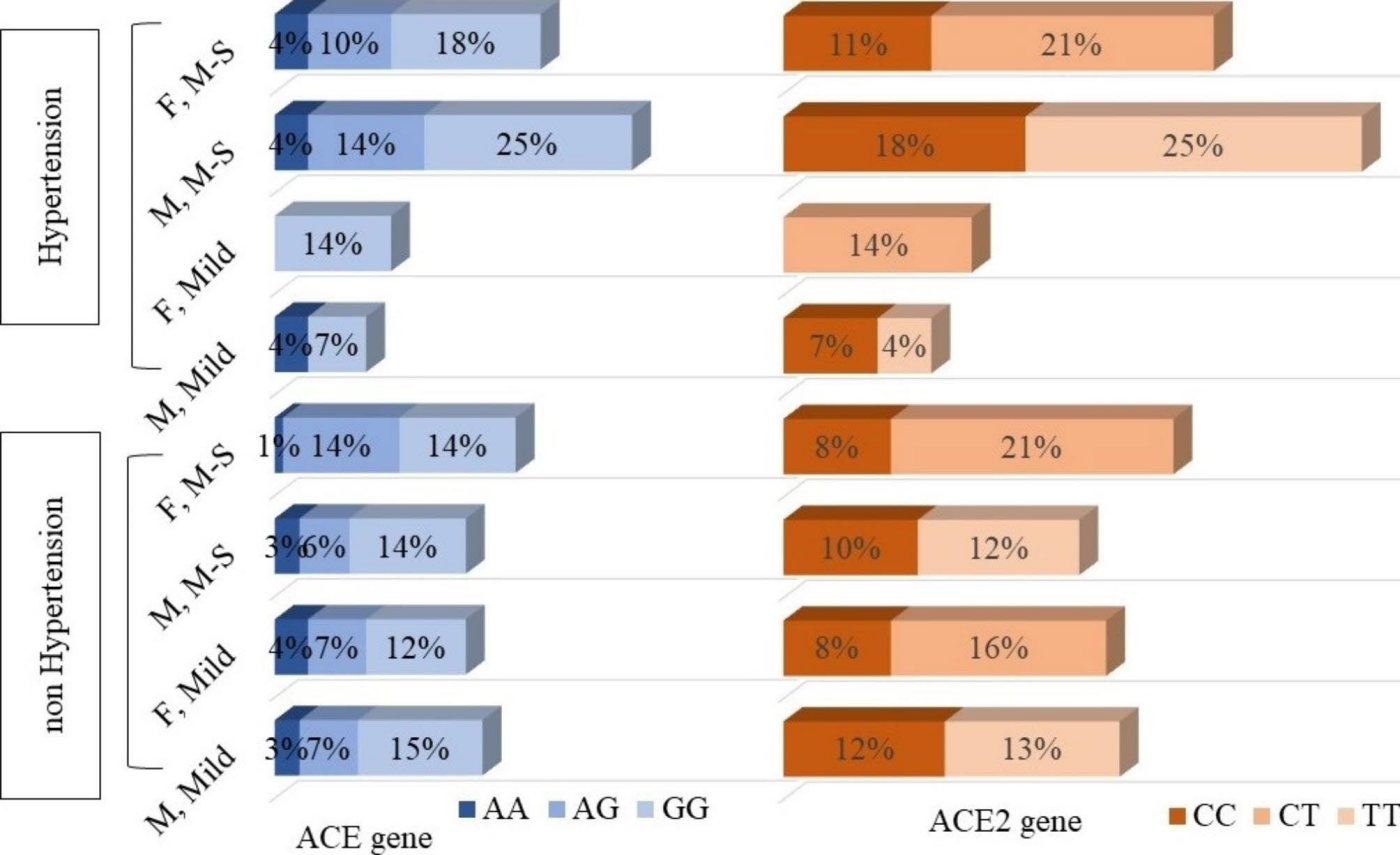
The effect of genetic variations on hypertension comorbidity, sex, and the severity of COVID-19. The GG and TT genotype groups in men with hypertension comorbidity had the highest percentage of moderate-to-severe COVID-19. F (Female), M (Male), M-S (Moderate-to-Severe)

We noted that 28 (29%) participants had hypertension, and 67 (70%) were without hypertension. An assessment of the effect of genetic variations on hypertension, sex, and the severity of COVID-19 is presented in Fig. 2. In the hypertension group, men with hypertension comorbidity and GG and TT genotypes tended to have moderate-to-severe symptoms (25%). On the other hand, the non-hypertensive group comprised mostly of women without hypertension comorbidity or with CT genotype.

## Discussion

Several studies have investigated the role of *ACE* and *ACE2* gene polymorphisms in COVID-19 susceptibility and disease severity [13–15]. ACE2 is a SARS-CoV-2 receptor, while ACE plays a role in blood pressure regulation. Functional variants that increase *ACE* and *ACE2* gene expression were thought to cause high viral binding to membrane sites, increasing carrier susceptibility to infection. Our previous study concluded that the rs4331 genotype showed consistent results, i.e., allele A was always found in samples with deletions and allele G with insertions (unpublished observations). These results confirm that rs4331 can be used to detect I/D polymorphisms. Our results show that the COVID-19 group tended to carry the GG genotype in the *ACE* gene and the CT genotype in *ACE2*. A study by Jacob (2021) found that the insertion of *ACE* gene increases the expression level of *ACE2* [16]. Increased expression of ACE2 may increase the risk of viral infection and the detrimental effects of *ACE*2 on the lungs and other organs. At the same time, it increases ACE activity in the Ang II/AT1R response [17].

The *ACE2* gene in the X chromosome may be detrimental in men because it carries only one copy of the X-linked *ACE2* gene. However, our study found a more significant proportion of women than men in the COVID-19 group and non-COVID-19. In addition, we found that carriers of the *ACE2* genotype were more susceptible to COVID-19 events. In the COVID-19 group, we found that CC carriers tend to be male, while women tend to be CT genotype carriers. In addition, we only found the TT genotype in males. This result aligns with a study by Patel (2012), which showed that males were dominant carriers of CC, and the proportion of TT genotypes was higher in males than in females [18]. In addition, Suleiman (2021) also concluded that the dominant Asian female population carries the CC or CT genotype without appreciable differences between males and females, compared to those observed in Caucasians [19].

Mutations in *ACE2* and *ACE* can cause an increase in serum levels and expression of ACE2 [20]. Kamyshnyi (2020) stated that men have higher ACE2 expression levels than women, and Asian populations have higher ACE2 transcript levels than Caucasian and African populations [21]. RAS imbalance due to increased ACE2 expression in the lungs facilitates inflammation and coagulation processes due to Ang-II overproduction and Ang- [1–7] deficiency. On the other hand, SARS-CoV-2 has an intrinsically high affinity for the ACE2 receptor, and mild or moderate ACE2 deficiency cannot play a protective role in host defense against viral invasion [17, 22]. Our results show that hypertension comorbidity in men is associated with a greater severity of COVID-19-induced ACE2 deficiency.

The SNPs rs4331 of the *ACE* and rs2074192 of the *ACE2* genes investigated in our study were in exonic (synonymous) and intronic positions. However, mutations occur in introns that alter mRNA splicing and affect gene expression and protein levels at ACE2 levels [23, 24]. As observed in our study, the genotype of the mutant in the *ACE* gene, i.e., GG and in the *ACE2* genes, i.e., TT for men and CT for women, are associated with the risk prevalence and severity of SARS-CoV-2 infection. These results are similar to those reported by Hubacek (2021) and Hamet (2021), in which they showed that mutations in the *ACE* and *ACE2* genes were directly proportional to the severity of COVID-19 [25, 26]. In addition, the effects of ACE and ACE2 on blood pressure were reported; ACE2 reduces the vasoconstrictor angiotensin II and activity of ACE/AT1R in RAS. In addition, it was assumed that genetic variations that increase the expression or activity of ACE and ACE2 might contribute to cardiovascular disease and SARS-CoV-2 infection [27].

However, the molecular mechanisms behind these observations are still minimally elucidated [21, 28]. We must also consider variants in other gene regions related to gene expression and protein level to also be involved in increasing severity of COVID-19. In conclusion, hypertension and mutations in the *ACE* (rs4331) and *ACE2* (rs2074192) genes can cause greater severity in COVID-19 patients; therefore, special attention and prompt treatment is needed in the clinical management of COVID-19 patients with hypertension.

### Limitations

Our study uses data from only two regions of Indonesia which were not affected by COVID-19 Delta cases. However, these two populations represent a geographic area with a high percentage of people with hypertension and deaths from COVID-19. Adjustments for potentially confounding variables, such as vaccination stage factors and hypertension therapy, may contribute to differences in COVID-19 severity profiles. Our results suggest that evaluating genetic variation in *ACE* and *ACE2* genes can be a useful new diagnostic approach for clinical assessment and risk management of COVID-19 with hypertension.

### Electronic supplementary material

Below is the link to the electronic supplementary material.

Supplementary Material 1

## Data Availability

The raw data supporting the conclusions of this article will be made available by the authors without undue reservation.

## Abbreviations

ACE: Angiotensin Converting Enzyme
ACE-2: Angiotensin Converting Enzyme-2
SARS-CoV-2: Severe acute respiratory syndrome coronavirus 2
COVID-19: Corona Virus Disease 2019
SNP: Single Nucleotide Polymorphism
RAS: Renin-Angiotensin System
I/D: Insertion/deletion
rtPCR: Real-time-polymerase chain reaction
