# Supplementary material for: Genotype variation of ACE and ACE2 genes affects the severity of COVID-19 patients
Source: BMC Res Notes. 2023 Sep 4;16:194. doi: 10.1186/s13104-023-06483-z (PMC10478384; doi:10.1186/s13104-023-06483-z)
Supplement: Supplementary file 1 — Supplementary Material 1 [file 13104_2023_6483_MOESM1_ESM.docx]

Suppl. Table 1 Clinical characteristics of severity COVID-19 by sex

| **Variable** | **Mild** | | **p-value** | **Moderate-Severe** | | **p-value** |
| --- | --- | --- | --- | --- | --- | --- |
|  | **Male**  **n (%)** | **Female**  **n (%)** |  | **Male**  **n (%)** | **Female**  **n (%)** |  |
| Symptoms |  |  |  |  |  |  |
| Cough | 12 (52) | 11 (48) | 1.000 | 21 (54) | 18 (46) | 0.421 |
| Mucus | 4 (57) | 3 (43) | 0.500 | 4 (40) | 6 (60) | 0.389 |
| Fatigue | 11 (48) | 12 (52) | 1.000 | 8 (35) | 15 (65) | 0.127 |
| Fever History | 13 (50) | 13 (50) | 1.000 | 19 (58) | 14 (42) | 0.205 |
| Diarrhea | 2 (40) | 3 (60) | 0.500 | 0 (0) | 2 (100) | 0.255 |
| Anosmia | 12 (55) | 10 (45) | 0.751 | 1 (7) | 14 (93) | **0.000** |
| Nausea | 7 (39) | 11 (61) | 0.340 | 9 (39) | 14 (61) | 0.327 |
| Vomiting | 4 (50) | 4 (50) | 0.653 | 1 (11) | 8 (89) | **0.014** |
| Dyspnea | 4 (44) | 5 (56) | 0.500 | 18 (51) | 17 (49) | 0.858 |
| Headache | 6 (55) | 11 (45) | 0.201 | 9 (45) | 11 (55) | 0.858 |
| Myalgia | 11 (48) | 12 (52) | 1.000 | 15 (50) | 15 (50) | 1.000 |
